# Supplementary material for: Genetic Variation of Puccinia triticina Populations in Iran from 2010 to 2017 as Revealed by SSR and ISSR Markers
Source: J Fungi (Basel). 2023 Mar 22;9(3):388. doi: 10.3390/jof9030388 (PMC10056552; doi:10.3390/jof9030388)
Supplement: Supplementary file 1 [file jof-09-00388-s001.zip › Table S1 (3).pdf]

Supplementary Table S1. Leaf rust isolates collected from various hosts in Iran from 2010 to 2017 and virulence phenotypes as determined by pathogenicity test on Tatcher's differential near-isogenic lines of wheat (adapted from [55])

Isolates	Year	Province	Host	Pathotypes
89.01	2010	Khuzestan	bread wheat	FDTTL
89.02	2010	Khuzestan	bread wheat	FBMTQ
89.05	2010	Khuzestan	bread wheat	FDTSQ
89.07	2010	Khuzestan	bread wheat	FDTTQ
89.13	2010	Khuzestan	bread wheat	FBMTQ
89.14	2010	Khuzestan	bread wheat	FDTSQ
89.19	2010	Khuzestan	bread wheat	FDTTQ
89.20	2010	Khuzestan	bread wheat	FBMNQ
89.24	2010	Ilam	bread wheat	FDRNQ
89.27	2010	Mazandaran	bread wheat	FDKRQ
89.28	2010	Kurdistan	bread wheat	CDKRQ
89.29	2010	Khuzestan	bread wheat	FDKNQ
89.30	2010	Khuzestan	bread wheat	FBHMQ
89.31	2010	Khuzestan	bread wheat	FDKNQ
89.32	2010	Khuzestan	bread wheat	FBMTQ
89.33	2010	Khorasan Razavi	bread wheat	FDPNQ
89.34	2010	Khorasan Razavi	bread wheat	FBPMQ
89.36	2010	Khorasan Razavi	bread wheat	FDTTQ
89.40	2010	Hamedan	bread wheat	CDKRQ
89.44	2010	Lorestan	bread wheat	FDRNQ
90.01	2011	Khuzestan	bread wheat	DDPRQ
90.02	2011	Khuzestan	bread wheat	DDPRQ
90.03	2011	Golestan	bread wheat	FFFLQ
90.04	2011	Mazandaran	bread wheat	FDKNQ
90.05	2011	Mazandaran	bread wheat	FDFLQ
90.06	2011	Mazandaran	bread wheat	FDKNQ
90.07	2011	Kurdistan	bread wheat	FDKPQ
90.08	2011	Kurdistan	bread wheat	FDFQQ
90.10	2011	Kurdistan	bread wheat	FDKPQ
90.13	2011	Ardabil	bread wheat	FDPNQ
90.14	2011	Ardabil	bread wheat	FDPNQ
90.17	2011	Golestan	bread wheat	CDDLB
90.18	2011	Mazandaran	bread wheat	FDTNQ
90.19	2011	Mazandaran	bread wheat	FDKPQ
90.20	2011	Mazandaran	bread wheat	FDKPQ
90.21	2011	Mazandaran	bread wheat	FDKNQ
90.25	2011	Mazandaran	bread wheat	FDKPQ

Supplementary Table S1. Continued

Isolates	Year	Province	Host	Pathotypes
90.26	2011	Ardabil	bread wheat	FDTNQ
90.27	2011	Ardabil	bread wheat	FDTNQ
90.28	2011	Ardabil	bread wheat	FDPNQ
90.9	2011	Khorasan Razavi	bread wheat	FDPNQ
91.02	2012	Mazandaran	bread wheat	FDTNQ
91.01	2012	Fars	bread wheat	FFFRQ
91.11	2012	Khuzestan	bread wheat	FKFFQ
91.03	2012	Mazandaran	bread wheat	FKTMQ
91.04	2012	Fars	bread wheat	FFFQQ
91.12	2012	Fars	bread wheat	FFFRQ
91.14	2012	Khuzestan	bread wheat	FDKLQ
91.15	2012	Golestan	bread wheat	FDKLQ
91.16	2012	Golestan	bread wheat	FFKRQ
91.18	2012	Mazandaran	bread wheat	FFKRQ
91.9	2012	Mazandaran	bread wheat	BDKQQ
91.22	2012	Khorasan Razavi	bread wheat	BDKQQ
91.23	2012	Khorasan Razavi	bread wheat	CDKQQ
91.24	2012	Khorasan Razavi	bread wheat	CDKQQ
91.25	2012	Khorasan Razavi	bread wheat	CDKQQ
91.26	2012	Khorasan Razavi	bread wheat	CDKQQ
91.27	2012	Khorasan Razavi	bread wheat	CDKQQ
91.34	2012	Lorestan	bread wheat	FKTMQ
92.01	2013	Mazandaran	bread wheat	FDKLQ
92.02	2013	Golestan	bread wheat	CDHLQ
92.03	2013	Golestan	bread wheat	FDKMQ
92.04	2013	Golestan	bread wheat	BDKLQ
92.05	2013	Golestan	bread wheat	FDKRQ
92.07	2013	Mazandaran	bread wheat	FPKNQ
92.08	2013	Mazandaran	bread wheat	FDKLQ
92.09	2013	Mazandaran	bread wheat	FDKPQ
92.10	2013	Mazandaran	bread wheat	FTKNQ
92.12	2013	Mazandaran	bread wheat	FDHLQ
92.15	2013	Mazandaran	bread wheat	CDHLQ
92.18	2013	Mazandaran	bread wheat	CDHLQ
92.19	2013	Golestan	bread wheat	FDKTQ
92.20	2013	Golestan	bread wheat	FJHPQ
92.21	2013	Golestan	bread wheat	FJHPQ
92.22	2013	Golestan	bread wheat	FHTQQ

Supplementary Table S1. Continued

Isolates	Year	Province	Host	Pathotypes
92.23	2013	Golestan	bread wheat	FHTQQ
92.24	2013	Golestan	bread wheat	FDKMQ
92.26	2013	Golestan	bread wheat	FDKRQ
93.01	2014	Khuzestan	bread wheat	CDRLQ
93.02	2014	Khuzestan	bread wheat	CBMLQ
93.03	2014	Lorestan	bread wheat	FGCLQ
93.04	2014	Mazandaran	bread wheat	FGCLQ
93.05	2014	Mazandaran	bread wheat	FDTLQ
93.06	2014	Mazandaran	bread wheat	FDKPQ
93.07	2014	Mazandaran	bread wheat	FDHTQ
93.08	2014	Mazandaran	bread wheat	FDKTQ
93.09	2014	Mazandaran	bread wheat	FDPLL
93.10	2014	Mazandaran	bread wheat	FDTLQ
93.11	2014	Mazandaran	bread wheat	FDHLQ
93.12	2014	Lorestan	bread wheat	FGCLQ
93.13	2014	Lorestan	bread wheat	FGCLL
93.14	2014	Lorestan	bread wheat	FDTNQ
93.15	2014	Lorestan	bread wheat	FDTNQ
93.16	2014	Lorestan	bread wheat	FGCLL
93.17	2014	Mazandaran	bread wheat	FJPSQ
93.18	2014	Mazandaran	bread wheat	FDKRQ
93.19	2014	Mazandaran	bread wheat	FNMLS
93.20	2014	Mazandaran	bread wheat	FNMLS
93.21	2014	Mazandaran	bread wheat	FNMLS
93.22	2014	Ardabil	bread wheat	FDTLQ
93.23	2014	Lorestan	bread wheat	FDTNQ
93.24	2014	Ardabil	bread wheat	CDFMQ
93.26	2014	Ardabil	bread wheat	CDFMQ
93.32	2014	Golestan	bread wheat	DJTRQ
93.33	2014	west Azerbaijan	bread wheat	FDTSQ
93.36	2014	west Azerbaijan	bread wheat	FDTSQ
93.37	2014	west Azerbaijan	bread wheat	FDTTQ
93.39	2014	East Azerbaijan	bread wheat	FDTTQ
93.40	2014	East Azerbaijan	bread wheat	FGQRS
93.41	2014	East Azerbaijan	bread wheat	FDTTQ
93.42	2014	Zanjan	bread wheat	FDTSQ
93.43	2014	Mazandaran	bread wheat	FDKTQ
93.44	2014	Ardabil	bread wheat	FJPSQ
93.45	2014	Ardabil	bread wheat	FJPSQ

Supplementary Table S1. Continued

Isolates	Year	Province	Host	Pathotypes
93.46	2014	Ardabil	bread wheat	FSTTS
93.A1	2014	Kerman	bread wheat	FDKPQ
93.A2	2014	Kerman	bread wheat	FDTNQ
93.A3	2014	Kerman	bread wheat	FDTTQ
93.A3.1	2014	Kerman	bread wheat	FDKLQ
93.A4	2014	Kerman	bread wheat	FDTTQ
94.11	2015	Mazandaran	bread wheat	BKKNG
94.12	2015	Mazandaran	bread wheat	BKKNG
94.15	2015	Mazandaran	bread wheat	CSKPQ
94.18	2015	Khuzestan	bread wheat	BDTPG
94.19	2015	Khuzestan	bread wheat	CDTPG
94.21	2015	Khuzestan	bread wheat	BDTPG
94.28	2015	Khuzestan	bread wheat	FKTNQ
94.27	2015	Mazandaran	bread wheat	FJKNQ
94.42	2015	Mazandaran	bread wheat	FJKNQ
94.47	2015	Mazandaran	bread wheat	CJTTG
94.48	2015	Mazandaran	bread wheat	CJTTG
95.04	2016	Khuzestan	durum wheat	FJTNQ
95.08	2016	Khuzestan	barley	FKTNQ
95.08.1	2016	Khuzestan	barley	FKTPQ
95.11	2016	Khuzestan	durum wheat	FJTSQ
95.12.1	2016	Khuzestan	triticale	LJKQS
95.12.2	2016	Khuzestan	triticale	LJKQS
95.12.3	2016	Khuzestan	triticale	LJKQS
95.12.4	2016	Khuzestan	triticale	LJKQS
95.12.5	2016	Khuzestan	triticale	LJKQS
95.14	2016	Khuzestan	durum wheat (Shabrang)	FJKRQ
95.14.1	2016	Khuzestan	durum wheat (Shabrang)	FJKRQ
95.17	2016	Khuzestan	barley	FJTTQ
95.17	2016	Khuzestan	barley	FJTTQ
95.17.1	2016	Khuzestan	barley	FKTTQ
95.20	2016	Khuzestan	durum wheat (Shabrang)	CDKSR
95.21	2016	Khuzestan	bread wheat	FKTTQ
95.21.1	2016	Khuzestan	bread wheat	FKKTQ
95.22	2016	Khuzestan	oat	DKTSS
95.22.1	2016	Khuzestan	oat	DKTSS

Supplementary Table S1. Continued

Isolates	Year	Province	Host	Pathotypes
95.22.2	2016	Khuzestan	oat	DKTSS
95.22.3	2016	Khuzestan	oat	DKTSS
95.22.4	2016	Khuzestan	oat	DKTSS
95.23.1	2016	Ilam	durum wheat (Karkhe)	FJKPQ
95.25	2016	Ilam	bread wheat	FFKTQ
95.26	2016	Ilam	durum wheat	FDCQS
95.29	2016	Mazandaran	bread wheat	DKKSQ
95.30	2016	Mazandaran	bread wheat	NJKSQ
95.33	2016	Mazandaran	bread wheat	FDTTQ
95.33.1	2016	Mazandaran	bread wheat	PKTSS
95.36	2016	Ardabil	bread wheat	FKTTQ
95.37	2016	Ardabil	bread wheat	PJTSQ
95.38	2016	Lorestan	wild barley	PJKTQ
95.39	2016	Lorestan	bread wheat	FDKTQ
95.40	2016	Lorestan	bread wheat	FKTTQ
95.43	2016	Khorasan Razavi	bread wheat	PKTTS
95.44	2016	Lorestan	bread wheat	FDKPQ
95.51	2016	Ardabil	bread wheat	PJTSQ
95.52	2016	Ardabil	bread wheat	FDTTQ
95.61	2016	Ardabil	bread wheat	PJKTS
B.96.1	2017	Fars (Bamu National Park)	wild barley	FKTNQ
B.96.2	2017	Fars (Bamu National Park)	wild barley	FKTNQ
B.96.3	2017	Fars (Bamu National Park)	wild barley	FKTNQ
MA.96.1	2017	Fars (Margoon Protected Region)	wild barley	FDFTR
MA.96.2	2017	Fars (Margoon Protected Region)	wild barley	FDFTQ
MA.96.3	2017	Fars (Margoon Protected Region)	wild barley	FDFTQ
