# Supplementary material for: Genetic Variation of Puccinia triticina Populations in Iran from 2010 to 2017 as Revealed by SSR and ISSR Markers
Source: J Fungi (Basel). 2023 Mar 22;9(3):388. doi: 10.3390/jof9030388 (PMC10056552; doi:10.3390/jof9030388)
Supplement: Supplementary file 1 [file jof-09-00388-s001.zip › Table S2 (2).pdf]

Supplementary Table S2. Host range and type of reactions on different hosts of leaf rust isolates from various hosts in Iran (adapted from [55]).

Isolate	Bread wheat (Bolani)		Dorum wheat (Yavaroos)		Dorum wheat (Karkhe)		Barley (Afzal)		Oat		Rye		Triticale	
	V[a]/A[b]	In[c]	V/A	In	V/A	In	V/A	In	V/A	In	V/A	In	V/A	In
89-36	V	3,4	A	0,;,N[d]	A	1,N	A	0,1	A	0	A	0	V	3,4
89-13	V	3,4	A	0,1	A	0,1	A	1,C[F]	A	0	A	0	V	4
89-27	V	3,4	A	0,;,N	A	0,1	A	0,1	A	0	A	0	V	3,4
89-24	V	3,4	A	0,;,N	A	0,1	A	1,C	A	0	A	0	V	4
90-8	V	3,4	A	1	A	0,;,C	A	;,C	A	0	A	;,2,C	A	1,2
90-4	V	3,4	A	;,1,C	A	;,1,C	A	;,C	A	0	A	1,C	V	2,3,C
90-17	V	3,4	A	1,C	A	0,;,C	A	;	A	0	A	;,C	V	2,3
90-9	V	3,4	A	;,1,C	A	1,C,N	A	;,C	A	0	A	2,C	V	2,3,4
90-1	V	3,4	A	;,C	A	1,C,N	A	;	A	0	A	2,C	V	3,4
91-1	V	3,4	A	;,1,C	A	1,C	A	;,1,C	A	0	A	;,1,C	V	2,3,4
91-4	V	3,4	A	1,;	A	;,1,C	A	;,1	A	0	A	1,C	V	2,3
91-15	V	3,4	A	1,C	A	;,1,C	A	;,1	A	0	A	;,1,C	V	3,4
91-12	V	3,4	A	C	A	;,C	A	;	A	0	A	1,C	V	3,4
91-18	V	3,4	A	;,1	A	;,1	A	;,1	A	0	A	C	V	2,3,C
92-1	V	3,4	A	1	A	1,C	A	;,1	A	0	A	;,C	V	3,4
92-2	V	3,4	A	;,1	A	;,1	A	;	A	0	A	C	V	3
92-24	V	3,4	A	1	A	1,C	A	;	A	0	A	C	V	3
93-A4	V	3,4	A	1,2	A	1	A	1,2	A	0	A	0,1	V	3,4
93-33	V	3,4	A	0,C	A	0,;	A	1,2,C	A	0	A	0,N	V	3,4
93-6	V	3,4	A	1	A	1	A	1,2,C	A	0	A	0,N	V	3,4
93-45	V	3,4	A	1	A	1	A	1,C	A	0	A	0	V	3,4
93-1	V	3,4	A	1,2	A	1,2	A	1,2	A	0	A	0,1	V	3,4
94-11	V	3,4	A	;,1,C	A	C12	A	;,1,C	A	0	A	;,1,C	V	2,3,3
94-18	V	3,4	A	;,1,C	A	C1	A	;,1	A	0	A	;,1,C	V	3,4
95-29	V	3,4	A	1	A	1,2	A	1	A	0	A	0	V	3,4
95-36	V	3,4	A	1	A	1,2	A	1,C	A	0	A	0	V	3,4
95-21	V	3,4	A	1,2	A	1,2	A	1,2,C	A	0	A	0,N	V	3,4
95-26-1	V	3,4	V	3	V	3,4	A	1,C	A	0	A	0	V	2,3,C
95-26-3	V	3	V	3,4	V	3,4	A	;,1	A	0	A	0	V	2,3,C
95-20-1	V	3	V	2,3	V	3,4	A	1,C	A	0	A	0	V	2,3
95-20-2	V	3	V	2,3	V	3,4	A	;,1	A	0	A	0	V	2,3,C
95-14-1	V	3	V	3	V	3,4	A	;	A	0	A	0	V	2,3
95-23-1	V	3	V	3	V	3,4	A	;	A	0	A	0	V	2,3

Supplementary Table S2. Continued

Isolate	Bread wheat (Bolani)		Dorum wheat (Yavaroos)		Dorum wheat (Karkhe)		Barley (Afzal)		Oat		Rye		Triticale	
	V[a]/A[b]	In[c]	V/A	In	V/A	In	V/A	In	V/A	In	V/A	In	V/A	In
95-23-3	V	3	V	3	V	3,4	A	;	A	0	A	0	V	2,3
95-11-2	V	3	V	3,4	V	3	A	1,C	A	0	A	0	V	2,3,C
95-4-1	V	3	V	3,4	V	3	A	1,C	A	0	A	0	V	2,C
95-22-1	V	3	A	;,1,C	A	1,C	A	1,C	V	3,4	A	0	V	2,3
95-22-3	V	3	A	1,C,N	A	1,N	A	1,0	V	3,4	A	1	V	2,3
95-12-1	V	3,4	A	0,N	A	0,N	A	1,0	A	0	A	1,2	V	3,4
95-12-2	V	3,4	A	1,N	A	1	A	1,0	A	0	A	1,2	V	3,4
95-38-1	V	3,4	A	1,N	V	2,3	A	1	A	0	A	1,N	V	3,4
96-B-1	V	3,4	A	0,N	A	N	A	1	A	0	A	1	V	3,4
96-B-2	V	3,4	A	1,C,N	A	1,N	A	1	A	0	A	1	V	4
96-Ma-1	V	3,4	A	0,1,N	V	2,3	A	1	A	0	A	1,N	V	3,4
96-Ma-2	V	3,4	A	1,N	V	2,3	A	1	A	0	A	1,N	V	3,4
95-8-1	V	3	A	0,1,N	V	2,3	V	3,4	A	0	A	1,N	V	3
95-8-2	V	3	A	1,C	A	1	V	3,4	A	0	A	1	V	3
94-22	V	3	A	1,N,C	A	1,N	V	3,4	A	0	A	1	V	3
95-17-1	V	3	A	0,1,N	V	2,3	V	3,4	A	0	A	1,N	V	3
95-17-3	V	3	A	1,N,C	A	1,N	V	3,4	A	0	A	1	V	3
