# Supplementary figures and images for: Genetic Variation of Puccinia triticina Populations in Iran from 2010 to 2017 as Revealed by SSR and ISSR Markers
Source: J Fungi (Basel). 2023 Mar 22;9(3):388. doi: 10.3390/jof9030388 (PMC10056552; doi:10.3390/jof9030388)

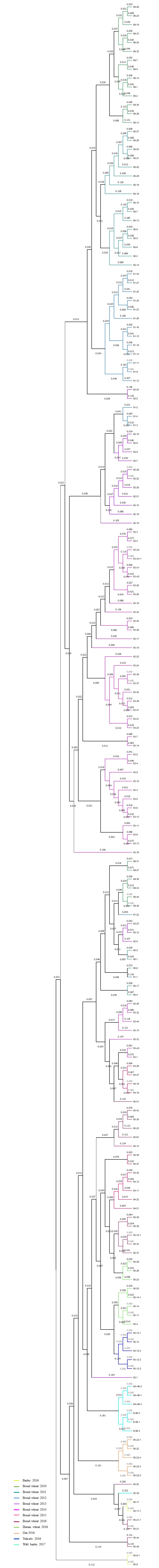

Supplement: Supplementary file 1 [file jof-09-00388-s001.zip › Figure S1.jpg]

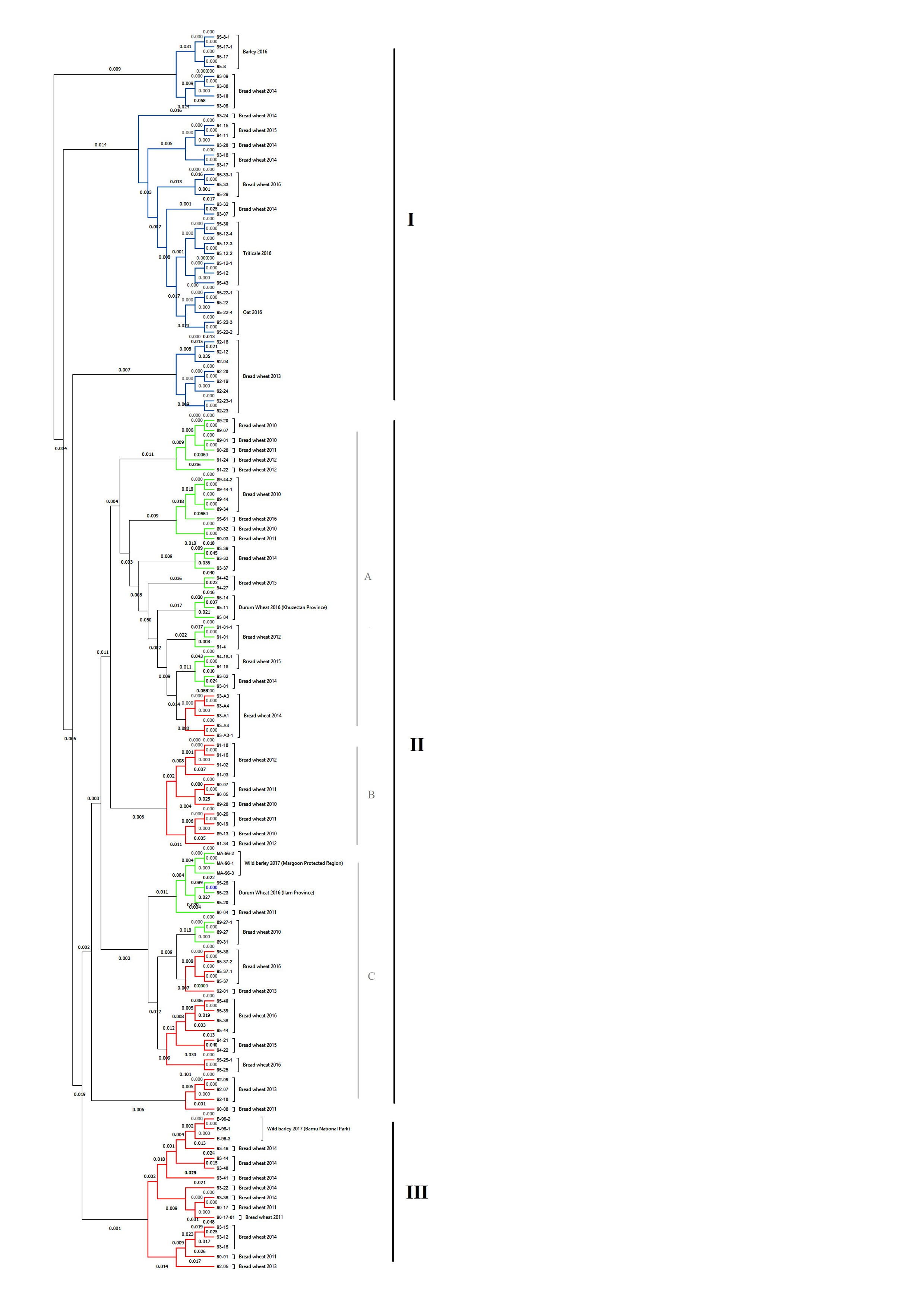

Supplement: Supplementary file 1 [file jof-09-00388-s001.zip › Figure S2.jpg]

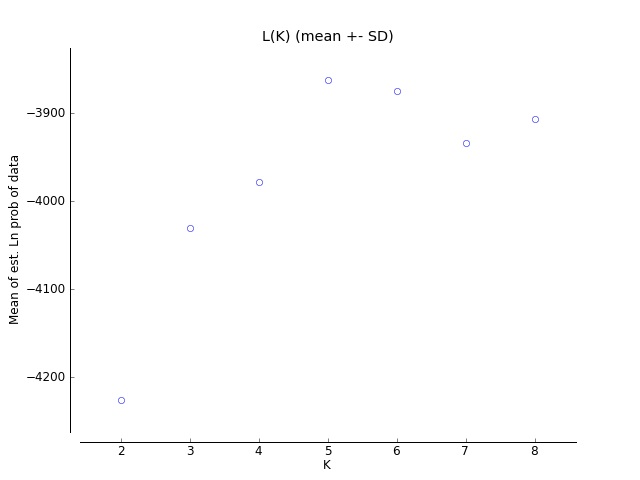

Supplement: Supplementary file 1 [file jof-09-00388-s001.zip › Figure S3.jpg]
